# Sustained *Wolbachia*-mediated blocking of dengue virus isolates following serial passage in *Aedes aegypti* cell culture

**DOI:** 10.1093/ve/vez012

**Published:** 2019-06-08

**Authors:** Cassandra Koh, Michelle D Audsley, Francesca Di Giallonardo, Emily J Kerton, Paul R Young, Edward C Holmes, Elizabeth A McGraw

**Affiliations:** 1 School of Biological Sciences, Monash University, Clayton, VIC, Australia; 2 Marie Bashir Institute for Infectious Diseases and Biosecurity, Charles Perkins Centre, School of Life and Environmental Sciences, The University of Sydney, Camperdown, NSW, Australia; 3 Sydney Medical School, The University of Sydney, Camperdown, NSW, Australia; 4 The Kirby Institute, School of Medical Sciences, University of New South Wales, Sydney, NSW, Australia; 5 Australian Infectious Diseases Research Centre, School of Chemistry and Molecular Biosciences, The University of Queensland, St Lucia, QLD, Australia; 6 Department of Entomology, Center for Infectious Disease Dynamics, Huck Institutes of the Life Sciences, Pennsylvania State University, State College, PA, USA

**Keywords:** dengue virus, *Wolbachia*, *Aedes aegypti*, evolution

## Abstract

*Wolbachia* is an intracellular endosymbiont of insects that inhibits the replication of a range of pathogens in its arthropod hosts. The release of *Wolbachia* into wild populations of mosquitoes is an innovative biocontrol effort to suppress the transmission of arthropod-borne viruses (arboviruses) to humans, most notably dengue virus. The success of the *Wolbachia*-based approach hinges upon the stable persistence of the ‘pathogen blocking’ effect, whose mechanistic basis is poorly understood. Evidence suggests that *Wolbachia* may affect viral replication via a combination of competition for host resources and activation of host immunity. The evolution of resistance against *Wolbachia* and pathogen blocking in the mosquito or the virus could reduce the public health impact of the symbiont releases. Here, we investigate if dengue 3 virus (DENV-3) is capable of accumulating adaptive mutations that improve its replicative capacity during serial passage in *Wolbachia w*Mel-infected cells. During the passaging regime, viral isolates in *Wolbachia*-infected cells exhibited greater variation in viral loads compared to controls. The viral loads of these isolates declined rapidly during passaging due to the blocking effects of *Wolbachia* carriage, with several being lost all together and the remainder recovering to low but stable levels. We attempted to sequence the genomes of the surviving passaged isolates but, given their low abundance, were unable to obtain sufficient depth of coverage for evolutionary analysis. In contrast, viral loads in *Wolbachia*-free control cells were consistently high during passaging. The surviving isolates passaged in the presence of *Wolbachia* exhibited a reduced ability to replicate even in *Wolbachia*-free cells. These experiments demonstrate the challenge for dengue in evolving resistance to *Wolbachia*-mediated blocking.

## 1. Introduction

The endosymbiont *Wolbachia* is being released into wild populations of the mosquito vector *Aedes aegypti* throughout the tropics as a strategy to reduce the transmission of viruses to humans (Hoffmann et al. 2011; Walker et al. 2011). In *Ae. aegypti* infected with *Wolbachia*, the replication of DENV—as well as other medically important positive single-stranded RNA viruses such as chikungunya, Zika, and yellow fever—is reduced, leading to the potential interruption in pathogen transmission (Walker et al. 2011; Van den Hurk et al. 2012; Dutra et al. 2016). A successful deployment in Townsville, Australia, has so far resulted in persistently high *Wolbachia* infection frequencies across the local mosquito population and elimination of domestic dengue transmission (O'Neill et al. 2018). Other studies of efficacy are underway in Southeast Asia and Latin America. The long-term stability of *Wolbachia*-mediated pathogen blocking in the field will be dependent on the complex selection pressures acting via the genomes of the three relevant organisms: *Wolbachia*, DENV, and the mosquito. Evolution of resistance either against *Wolbachia* or its effects in the viral or mosquito genomes could lead to reduced efficacy.

The mechanistic nature of *Wolbachia*-mediated blocking is currently poorly defined. The originally proposed putative mechanism was that of innate immune priming, whereby the presence of *Wolbachia* heightens the basal immune response of the insect, improving the insect’s ability to control any secondary pathogen it encounters. This theory originated from transcriptional profiling data in the mosquito demonstrating that *Wolbachia* induces transcription of the canonical innate immune pathways (Kambris et al. 2009, 2010; Moreira et al. 2009). Later work, including some comparative studies in the naturally infected *Drosophila melanogaster* and in *Ae. aegypti* (Rances et al. 2012; Terradas, Joubert, and McGraw 2017), demonstrated that the priming of these core pathways could not explain the entirety of the effect. Other studies have found evidence of competition between the symbiont and viruses for intracellular space (Moreira et al. 2009; Rainey et al. 2016) and cholesterol (Caragata et al. 2013), and still others cite the involvement of reaction oxygen species (Pan et al. 2012; Wong, Brownlie, and Johnson 2015) or *Wolbachia*-directed control of host gene expression by small RNA production (Zhang et al. 2013; Mayoral et al. 2014; Asad et al. 2018). Commonly, the strength of blocking is positively correlated with *Wolbachia* density (Osborne et al. 2009, 2012; Frentiu et al. 2010; Walker et al. 2011; Lu et al. 2012; Chrostek et al. 2013; Amuzu and McGraw 2016).

In wild mosquito populations where *Wolbachia* have been released for biocontrol and subsequently spread to high frequencies, a virus will be under strong pressure to increase its replicative fitness in the presence of the symbiont (Bull and Turelli 2013). Replication of the small, positive, single-stranded RNA genome of DENV is highly error prone, as it depends upon an RNA-dependent RNA polymerase that lacks proof-reading ability. As a consequence, the population of DENVs within a single host (human or insect) is highly genetically diverse and likely phenotypically heterogeneous (Wang et al. 2002; Parameswaran et al. 2017). Regardless, Bull and Turelli (2013) predicted that adaptive mutations allowing the virus to completely overcome *Wolbachia*-induced blocking would be difficult to achieve given the likely multifaceted nature of blocking mechanisms (Johnson 2015), coupled with the constraints of strong purifying selection necessarily resulting from replication in both vertebrate and invertebrate cells (Vasilakis et al. 2011).

Recent work has suggested that *Wolbachia* interference occurs at the level of intracellular replication and assembly (Welsch et al. 2009; Zhang et al. 2013; Rainey et al. 2016; Bhattacharya, Newton, and Hardy 2017; Asad, Parry, and Asgari 2018; Asad et al. 2018). Hence, any adaptive mutations in viral proteins that improve efficiency at these stages may represent an evolutionary solution to resistance against *Wolbachia*. Improved functions of certain viral proteins could also relax any competition between *Wolbachia* and DENV for host resources, such as lipids and intracellular space, reducing the impact of the blocking phenotype. For example, the structural capsid protein primarily packages the viral genome and acts as a chaperone to promote correct folding, but it has also been reported to bind to lipid droplets (Samsa et al. 2009; Byk and Gamarnik 2016). Lipid droplets are intracellular vesicles containing triacylglycerides and cholesterol (Ducharme and Bickel 2008). The presence of DENV capsid proteins increases the abundance of lipid droplets in infected cells (Samsa et al. 2009). Although the underlying mechanism is unknown, increased lipid droplet enrichment by the capsid protein could lead to a higher availability of lipid sources of energy for viral replication. Furthermore, DENV nonstructural proteins are involved in assembling membrane-bound viral replication complexes, derived from the endoplasmic reticulum (ER) and enriched in cholesterol (Muller and Young 2013; Xie et al. 2017). As *Wolbachia* and DENV are both intimately associated with and compete for ER membrane structures (Welsch et al. 2009; White et al. 2017), changes in viral nonstructural proteins that improve the efficiency of replication complex formation may help to overcome this competition. Given the complex nature of blocking mechanisms, adaptation may only be possible through multiple beneficial mutations in viral protein encoding genes.

A variety of studies have attempted to model the coevolution of this mosquito-symbiont-pathogen system (Lively et al. 2005; Vavre and Charlat 2012; Bull and Turelli 2013), but none have empirically examined the adaptive potential of DENV in the presence of *Wolbachia*. In this work, we sought to test if adaptive evolution in DENV can be experimentally induced *in vitro* in response to the presence of *Wolbachia.* To achieve this, DENV-3 viral isolates were serially passaged in three distinct *Ae. aegypti* cell lines, namely (1) a naïve *Wolbachia*-free line, (2) a tetracycline-cured *Wolbachia*-free line, and (3) a *Wolbachia*-infected line. During the regime, we transferred only viruses that were able to successfully infect the cell types, replicate, and escape into the surrounding medium. We expected to produce virus isolates that, after accumulating adaptive mutations, exhibited increased replicative fitness relative to both the ancestral virus and those isolates passaged in *Wolbachia*-free cells. We then compared the replicative fitness of the ancestral and passaged virus isolates in parallel in all three cell types and attempted to characterize the sequence diversity generated in the DENV isolates during the selection regime. Our results speak to the potential for DENV to adapt in response to *Wolbachia* in the short-term.

## 2. Materials and methods

### 2.1 Aag-2 cell lines

Three lines of Aag-2 cells were used in the virus evolution experiment: Aag-2, Aag-2Tet, and Aag-2*w*Mel. The Aag-2 cell lineage was derived from *Ae. aegypti* embryos and is susceptible to arthropod-borne virus (arbovirus) infections (Peleg 1968). Aag-2 cells naturally do not carry any *Wolbachia* infection. Aag-2*w*Mel cells were generated through transfection of the *w*Mel *Wolbachia* strain isolated from its natural host, *D. melanogaster* (Terradas, Joubert, and McGraw 2017). To create the Aag-2Tet cell line, Aag-2*w*Mel cells were cured of *Wolbachia* infection through treatment with the antibiotic tetracycline, as described in Terradas, Joubert, and McGraw (2017). All three cells lines were routinely maintained at 25 °C on complete medium consisting of equal parts Schneider’s medium and Mitsubishi and Maramorosch medium and supplemented with 10 per cent (v/v) heat-inactivated fetal bovine serum (FBS) (Life Technologies, Carlsbad, CA, USA) and 1 per cent (v/v) penicillin/streptomycin antibiotic (Life Technologies).

### 2.2 Virus

For this study, we used a DENV-3 isolated from Cairns (Queensland, Australia), during an outbreak in 2008/2009 (Ritchie et al. 2013). The strain had been passaged ten times post isolation in C6/36 *Aedes albopictus* cells maintained at 25 °C on RPMI 1640 medium supplemented with 10 per cent (v/v) FBS (Life Technologies), 1× GlutaMAX (Gibco), and 20 mM HEPES (Sigma-Aldrich, St Louis, MO, USA). To propagate sufficient virus for this experiment, monolayers of C6/36 cells in two T175 flasks at 80 per cent confluency were inoculated with DENV-3. Inoculated cells were then incubated in medium supplemented with 2 per cent (v/v) FBS (Life Technologies). At seven days post-inoculation, clarified cell culture medium from the two flasks were collected and pooled. The pooled virus concentration was 10^9^ genome copies per ml, as quantified via methods described below. This ancestral virus (passage 0, P_0_) was then stored at −80 °C in single-use aliquots for use in initial inoculation of the sequential passaging regime, repeated blocking tests, and replicative fitness assays.

### 2.3 Virus passaging

Six-well cell culture plates were seeded with Aag-2, Aag-2Tet, and Aag-2*w*Mel cells at a density of 1 × 10^6^ cells per well. Each well was inoculated with 2 ml of P_0_ and passaged five days later. In total, twelve virus lines were generated and transferred for nine sequential passages with five days of growth in between: three passaged in Aag-2 cells, three in Aag-2Tet cells, and six in Aag-2*w*Mel cells. Twice as many Aag-2*w*Mel lines were passaged to account for potential loss of virus due to complete blocking by *Wolbachia*. At each sequential passaging, cell supernatants were collected and clarified via centrifugation at 200 × g for 10 min. A total of 1 ml of this viremic supernatant was transferred to a fresh set of uninfected cells, followed by an addition of 800 µl of complete medium containing 2 per cent (v/v) FBS per well. The remaining supernatants were stored at −80 °C for later RNA extraction and sequencing. At every passage, *Wolbachia* was quantified and the uninfected status of Aag-2 and Aag2Tet cells was verified using the methods below. The DENV-blocking capability of the *w*Mel cells relative to Aag-2 and Aag2Tet cells was also confirmed at each passage in a parallel assay by inoculating all three cell lines with P_0_. At five days post-inoculation, supernatants from this blocking test and from passaged isolates were sampled, and DENV load was quantified using methods described below.

### 2.4 *Wolbachia* density quantification

A multiplex quantitative real-time polymerase chain reaction (qRT-PCR) was used to detect the presence of *Wolbachia* and quantify its density in our cell lines. This method has been previously described in Ferguson et al. (2015). In our study, all qRT-PCR assays were performed with a LightCycler 480 instrument II (Roche, Basel, Switzerland). Sample DNA was prepared by resuspending cell pellets in 50 µl of extraction buffer (10 mM Tris pH 8.2, 1 mM EDTA, 50 mM NaCl, and 1.25% (v/v) proteinase K [Bioline, Memphis, TN]) and incubating this suspension in a thermal cycler for 5 min at 56 °C, 5 min at 95 °C, and held at 4 °C (Yeap et al. 2014). A total of 1 µl of DNA sample was used in a single qRT-PCR reaction combined with 5 µl of 2× LightCycler 480 probes master mix (Roche), 0.25 µM of *rps17* forward and reverse primers, 0.1 µM of *rps17* probe, 0.3 µM of *WD0513* forward and reverse primers and probes, and RNase-free water to make up a total volume of 10 µl. Primer and probe sequences are shown in Supplementary Table S1. Cycling conditions were as follows: initial denaturation at 95 °C for 5 min; 45 cycles of amplification consisting of 95 °C for 10 s, 60 °C for 15 s, and 72 °C for 1 s; and a cooling step at 40 °C for 10 s. Each sample was run in triplicate. Relative *Wolbachia* density was calculated as the ratio of the *Wolbachia* gene *WD0513* to the housekeeping *Ae. aegypti* ribosomal protein gene *rps17* by using the Q-gene method (Simon 2003).

### 2.5 Fluorescent in situ hybridization for Wolbachia

The presence of *Wolbachia* in cells was also validated through fluorescent *in situ* hybridization (FISH) at every passage of the virus evolution experiment. Aag-2, Aag-2Tet, and Aag-2*w*Mel cells were seeded onto Nunc Lab-Tek 8-well chamber slides (Thermo Fisher Scientific, Waltham, MA, USA). When the cells were confluent, the cell culture medium was removed and cells were fixed, hybridized with rhodamine-labeled probes specific to *Wolbachia* 16s rRNA and DAPI, then mounted as per previously published methods (Moreira et al. 2009). Images were captured using a Leica DM compound fluorescent microscope with a Leica DC300 camera through the appropriate channels, keeping exposure times uniform across samples.

### 2.6 Viral quantification

The viral genome copy number in cell medium supernatant was quantified using TaqMan fast virus 1-step master mix (Applied Biosystems, Foster City, CA, USA). For every supernatant sample to be quantified, 10 µl of supernatant was mixed with extraction buffer in a 1:1 ratio and incubated as per above. Each qRT-PCR reaction consisted of 2.5 µl of 4× master mix, 0.5 µM of DENV forward and reverse primers, 0.25 µM of probe (Warrilow et al. 2002), 2 µl of viral RNA sample in extraction buffer, and RNase-free water to make up a total volume of 10 µl. Primer and probe sequences are shown in Supplementary Table S1. Cycling conditions were as follows: reverse transcription at 50 °C for 10 min; denaturation at 95 °C for 20 s; 50 cycles of amplification consisting of 95 °C for 3 s, 60 °C for 60 s, and 72 °C for 1 s; and a cooling step at 40 °C for 10 s. Absolute DENV-3 copy numbers were obtained by extrapolating to a standard curve of DENV genome copies ranging from 10^8^ to 10^1^ in a tenfold serial dilution, created as described in Ye et al. (2014). Each sample was run in triplicate. Copy number was expressed as genome copies per µl of supernatant. Loads were compared using analysis of variance (ANOVA) and post hoc tests performed on log-transformed values with GraphPad Prism 7 software (San Diego, CA, USA).

### 2.7 Replicative fitness assay

After experimental evolution through sequential passaging, each of the twelve virus lines was assayed for their replicative fitness in Aag-2, Aag-2Tet, and Aag-2*w*Mel cells (*n* = 5 per isolate per cell line). Ninety-six-well plates were seeded with 5 × 10^4^ cells per well with 200 µl of complete medium with 10 per cent (v/v) FBS (Life Technologies) and incubated for two days prior to inoculation. Cells were washed once with 200 µl of sterile 1× PBS before being inoculated with 1.5 × 10^4^ genome copies per well, followed by incubation for 2 h at 25 °C with rocking at every 15  min. Viral inoculants were then removed and 200 µl of complete medium with 2 per cent (v/v) FBS (Life Technologies) was added to each well. Post-inoculation, 10 µl of cell medium was sampled every day for seven days. Virus accumulation curves were obtained by sampling 10 µl of cell medium every day for seven days and quantifying number of virus genome copies via qRT-PCR as per methods described above.

### 2.8 RNA isolation and sequencing

Material was collected for RNA sequencing at generations 0, 5, and 9 for the *w*Mel-infected and tetracycline-cured control cells. Viral RNA in the cell medium supernatant was extracted using the TRIzol LS reagent (Invitrogen, Carlsbad, CA, USA) according to manufacturer’s protocol. Each sample of viral RNA was solubilized in 44 µl of RNase-free water. To each sample, 1 µl of DNase I recombinant enzyme (Roche) and 5 µl of buffer was added and incubated at 37 °C for 50 min to ensure no contamination of genomic DNA. To clean up the isolated RNA for sequencing use, we used an RNeasy kit (Qiagen, Hilden, Germany) according to the manufacturer’s protocol and eluted purified RNA into 30 µl of RNase-free water. Samples were prepared with the Illumina whole-transcriptome library prep kit with Ribo-Zero Gold rRNA depletion, and sequencing was performed on the Illumina NextSeq 500 platform at the Australian Genome Research Facility (AGRF).

### 2.9 Sequence data analysis

Illumina paired-reads were assembled in Geneious 11.1.3 (https://www.geneious.com, last accessed 12 January 2019) to the DENV-3 reference genome (GenBank accession number JN406515). Poor quality reads were removed. The resulting assemblies were manually inspected for genome coverage and potential genetic variations within the virus population. Notably, 54,972,167 reads mapped to the reference DENV-3 genome for generation 0, with an average coverage of 375,511 reads per nucleotide position. No genetic difference was observed between the sequenced sample and the reference sequence. Thus, the DENV-3 sequence was suitable to be used as reference for generations 5 and 9. However, read count and, thus, coverage, for these two generations was very low: less than 300 reads assembled in total for either generation. This reduced the median coverage to two reads per nucleotide position across the genome, although parts of the genome were not covered with any reads. Such limited coverage prevented analysis on virus genetic diversity or evolution in the genomes of the Aag-2*w*Mel-passaged isolates, and thus isolates from *Wolbachia*-free control lines were not sequenced. PCR amplification of the genome to increase material for sequencing was not considered as the very low viral load would make amplification of full genomes challenging. Additionally, we mapped reads to the cell fusing agent virus (CFAV) reference genome to check for its presence as this insect-specific flavivirus is known to persistently infect Aag2 cells and enhance DENV infection (Stollar and Thomas 1975; Zhang et al. 2017). Assembly was conducted as described for DENV-3 and also using Bowtie 2 v2.3.4.3 (Langmead et al. 2018).

## 3. Results

### 3.1 Viral loads throughout serial passaging

**Figure 1.**
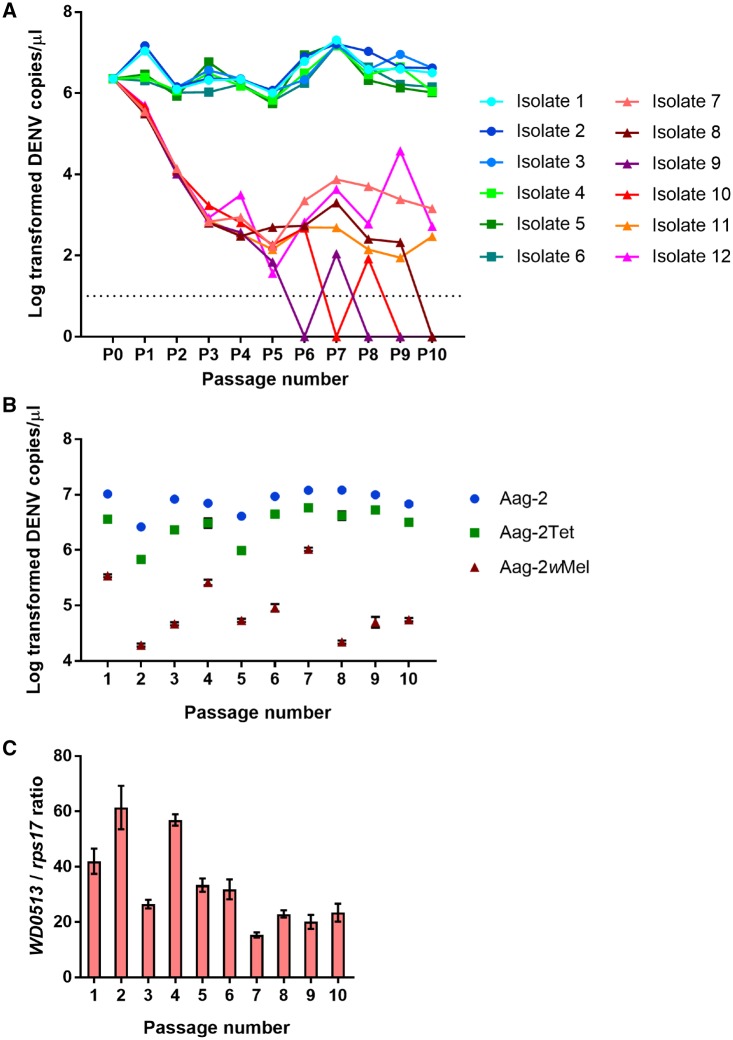
Sequential passaging of twelve DENV-3 isolates in Aag-2, Aag-2Tet, and Aag-2*w*Mel cells. (A) Log_10_ transformed viral genome copies of the twelve isolates at every passage, as measured via qRT-PCR. Blue-themed circles denote Aag-2-passaged isolates (1–3). Green-themed squares denote Aag-2Tet-passaged isolates (4–6). Red-themed triangles denote Aag-2*w*Mel-passaged isolates (7–12). Data points at 0 log_10_ copies/µl indicate that viral loads were below detection limits, which are at ten genome copies/µl (dotted line). (B) Log_10_ transformed viral genome copies in three cell lines inoculated with P_0_ to verify *Wolbachia*-induced blocking phenotype at five days post-inoculation. Mean and SEM are shown in graphs (*n* = 5 per cell line). (C) *Wolbachia* densities in Aag-2*w*Mel cells at every passage measured as the ratio of *Wolbachia* gene *WD0513* to mosquito gene *rps1*7.

During serial passaging, Aag-2- and Aag-2Tet-passaged isolates showed little variation in viral loads, whereas variation among the viral loads of Aag-2*w*Mel-passaged isolates increased dramatically with higher passage numbers (Fig. 1A). Viral copy number of isolates passaged in Aag-2*w*Mel cells showed a decreasing trend from P_1_ but began to stabilize from P_4_ onward. At P_10_, three of these isolates were lost, whereas three others (isolates 7, 11, and 12) remained at 10^3^–10^4^ genome copies per µl. On average, the loads of Aag-2*w*Mel-passaged isolates at the end of serial passaging were 3–4 log_10_-folds lower than the levels of isolates passaged in *Wolbachia*-free cells.

To verify that the *Wolbachia*-induced blocking phenotype was present throughout the experiment, cells used at each passage were challenged in parallel with an aliquot of P_0_ at multiplicity of infection (MOI) of 0.1, and the viral copy number present in the supernatant was quantified at five days post-inoculation (Fig. 1B). Throughout the passaging regime, DENV-3 genome copies in Aag-2*w*Mel cells were always lower than those in Aag-2 or Aag-2Tet cells by at least one log_10_-fold, confirming the effect of the pathogen blocking phenotype (*P* < 0.0001, Tukey’s multiple comparison test). The reduced loads in Aag-2Tet cells relative to Aag-2 cells can be explained by adverse side effects from tetracycline treatment on mitochondrial function, potentially dampening viral replication efficiencies in these cells (Moullan et al. 2015). *Wolbachia* densities were quantified via qRT-PCR at every passage (Fig. 1C). While they vary substantially, high infection frequencies throughout were validated through FISH (Supplementary Fig. S1).

### 3.2 Replicative fitness following selection

To detect fitness gains as a result of serial passaging, we assessed the replicative fitness of the nine virus isolates that remained at P_10_ in Aag-2, Aag-2Tet, and Aag-2*w*Mel cells. As the viral copy number of Aag-2*w*Mel-passaged isolates (isolates 7, 11, and 12) was much lower than that of Aag-2- or Aag-2Tet-passaged isolates (isolates 1–6), all inocula were standardized to 1.5 × 10^4^ virus copies per 5 × 10^4^ cells for all isolates in this assay—the highest virus copy-to-cell ratio possible while maintaining consistency across all isolates. We compared the replication kinetics of passaged isolates to the ancestral virus in all three cell lines used in this study to detect any fitness gains obtained from serial passaging. Supernatants were collected at two-day intervals during the fitness assay, but due to the lack of virus replication in the Aag-2*w*Mel cells, only samples collected at six days post-infection were quantified via qRT-PCR.

**Figure 2.**
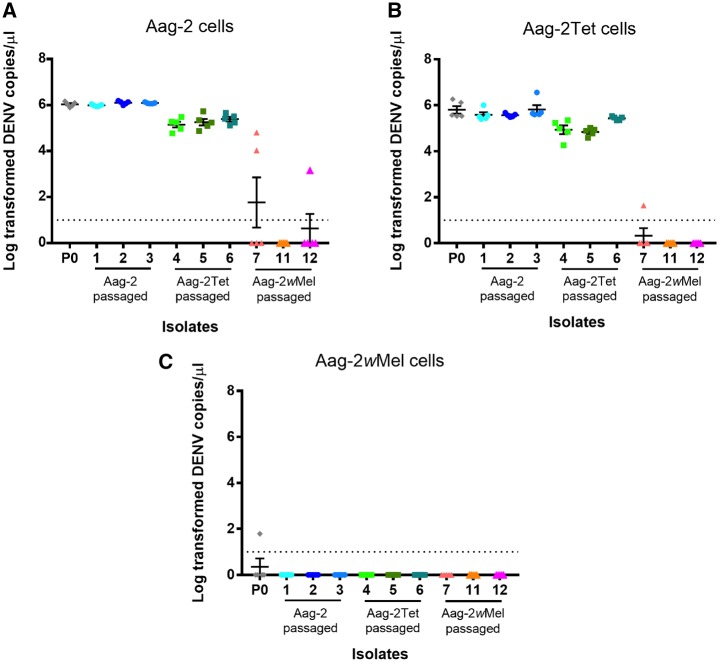
Replicative fitness assay of the nine remaining isolates following sequential passaging. Log_10_ transformed viral genome copy numbers in the supernatants of (A) Aag-2, (B) Aag-2Tet, and (C) Aag-2*w*Mel cells were quantified via qRT-PCR at six days post-inoculation by each isolate. Each data point represents one biological replicate. Data points at 0 log_10_ copies/µl indicate that viral copies were below detection limits at ten genome copies/µl (dotted line) and were excluded from data analysis. Mean and SEM are shown in graphs (*n* = 5 per isolate).

We did not find evidence of an increase in the replicative fitness relative to P_0_ in any of our passaged isolates, even when reinoculated onto the cell line in which they had been serially passaged. In Aag-2 and Aag-2Tet cells, the replication of Aag-2-passaged isolates was comparable to the P_0_ (Fig. 2A and B). Interestingly, Aag-2Tet-passaged isolates showed decreased replicative ability compared to P_0_ (*P* < 0.001, Dunnett’s multiple comparison test) (Table 1), with the exception of isolate 6 in Aag-2Tet cells. In Aag-2*w*Mel cells, none of the passaged isolates produced detectable viral copies (Fig. 2C).

**Table 1.** Summary of one-way ANOVAs and multiple comparisons tests comparing the replicative fitness of each passaged isolate to that of P_0_.

Test cell line	ANOVA *P*-value	Multiple comparisons test	*P*-value
Aag-2	<0.0001	P_0_ vs. isolate 1	n.s.
		P_0_ vs. isolate 2	n.s.
		P_0_ vs. isolate 3	n.s.
		P_0_ vs. isolate 4	0.0001
		P_0_ vs. isolate 5	0.0001
		P_0_ vs. isolate 6	0.0003
		P_0_ vs. isolate 7	0.0001
		P_0_ vs. isolate 12	0.0001
Aag-2Tet	<0.0001	P_0_ vs. isolate 1	n.s.
		P_0_ vs. isolate 2	n.s.
		P_0_ vs. isolate 3	n.s.
		P_0_ vs. isolate 4	0.0004
		P_0_ vs. isolate 5	0.0001
		P_0_ vs. isolate 6	n.s.
		P_0_ vs. isolate 7	0.0001

n.s., not significant.

The Aag-2*w*Mel-passaged isolates performed poorly in all tested cell lines. Very few biological replicates produced detectable viral loads, all of which were significantly lower than those of P_0_ (*P* < 0.0001, Dunnett’s multiple comparison test) (Table 1). Data points below the qRT-PCR detection limit were excluded from statistical analysis. The poor ability of these isolates to replicate in either *Wolbachia*-free cell line was surprising, as Aag-2- and Aag-2Tet-passaged isolates were able to produce replicated virions from similar inoculum concentrations. However, we believe the few Aag-2*w*Mel-passaged replicates with detectable viral loads (Fig. 2A and B) consisted of true products of replication during the assay and not remnants of the inoculum, as the supernatants of all replicates at one-day post-inoculation did not contain detectable numbers of genome copies (data not shown).

### 3.3 DENV-3 sequence diversity generated during the selection regime

Post-selection we prepared the following set of samples for sequencing; the ancestral P_0_ isolate and isolates 7, 11, and 12 (from Aag-2*w*Mel cell lines) at both P_5_ and P_9_. The resulting depth of sequencing coverage was very low in the passaged isolates: the total number of reads mapped to the DENV-3 genome averaged ∼290 across the passaged lines, compared to the ancestral isolate with ∼55,000. Approximately 80 per cent of the genome was only represented by 1x read coverage in the passaged isolates. Hence, these data reveal the rarity of virus in the culture and necessarily prevented statistical analysis of shifts in allele frequencies relative to the ancestor required to demonstrate evolutionary change. Thus, it is inconclusive whether evolution occurred in Aag-2*w*Mel-passaged isolates. To ensure that CFAV, which persistently infects Aag2 (Stollar and Thomas 1975), is not confounding DENV infection, we also assembled reads against CFAV reference genome. No contigs covering the CAFV genome were found, indicating that CFAV likely absent from these isolates.

## 4. Discussion

The higher variation in viral load in Aag-2*w*Mel-passaged isolates relative to those passaged in *Wolbachia*-free lines may reflect an underlying variation in fitness or random noise, which may in turn be linked to the substantial population bottlenecks during passage. A similar phenomenon was observed in foot-and-mouth-disease virus in which sequential population bottlenecks were experimentally induced during plaque-to-plaque transfers (Lazaro et al. 2003). This notwithstanding, the failure of viral copy numbers to revive substantially after their initial decline when passaged in Aag-2*w*Mel cells, the eventual loss of three of these isolates at P_10_, and the very low sequence coverage in the passaged isolates at P_9_ suggests that adaptive mutations against *Wolbachia*-induced blocking may be hard to achieve and maintain in viral populations. Indeed, the viral loads of Aag-2*w*Mel-passaged isolates by the end of serial passaging were 3–4 log_10_-folds lower than isolates passaged in *Wolbachia*-free cells. Assuming that the viral RNA polymerase error rate is constant regardless of cell line, the total opportunities for mutational events in these populations were therefore much reduced compared to *Wolbachia*-free controls, lessening the chance for an evolutionary solution to blocking. The lack of increased replicative fitness in the passaged isolates relative to P_0_, even when reinoculated into the original cell line in which they had been passaged, was unexpected given that recent studies have demonstrated that serial passaging of RNA viruses in a single host leads to rapid (<10 passages) fitness gains in said host (Coffey et al. 2008; Vasilakis et al. 2009; Grubaugh et al. 2015). Most of the Aag-2Tet-passaged isolates showed decreased replicative ability compared to P_0._ It is possible that the suboptimal conditions for viral replication as a side effect of tetracycline treatment (Moullan et al. 2015) induced some unexpected selective pressures, producing genotypes that are generally less fit. The Aag-2*w*Mel-passaged isolates performed poorly in all tested cell lines. In the *Wolbachia*-infected cell lines the inoculum may have been too low to recover in the presence of the symbiont. However, the inability of these isolates to replicate in *Wolbachia*-free cell lines was surprising and unlike that of the Aag-2- and Aag-2Tet-passaged isolates. This difference may suggest that while a subset of viruses was able to escape the *Wolbachia* effect, they may be less fit in a wildtype context.

It is possible that the lack of replication by Aag-2*w*Mel-passaged isolates in *Wolbachia*-free cells could be explained by a higher than expected proportion of defective virus genome copies being produced during the later stages of serial passaging, leading to an overestimation of inoculum size used in this fitness assay. To replicate, defective virus particles rely on the presence of coinfecting complete viral genome copies for functional complementation and hence may interfere with the replication of functional virus copies through competitive inhibition (Huang and Baltimore 1970; Stauffer Thompson, Rempala, and Yin 2009). They have been reported to exist in both *in vitro* and *in vivo* systems and are capable of being carried through many transmission cycles (Wang et al. 2002; Aaskov et al. 2006; Li et al. 2011; Juarez-Martinez et al. 2013). Our qRT-PCR method of quantifying viral load detects viral genome copies instead of infectious virions. Genome copies are often used as a proxy for infectious particles because the two are well correlated, but the presence of immature virus or defective genomes can inflate estimations of viral load by as much as 2–5 log_10_-folds (Choy et al. 2013). If Aag-2*w*Mel-passaged isolates contain a higher proportion of defective genome copies, the number of functional virus particles may be too low to cause infection even in *Wolbachia*-free cells. One way to validate this hypothesis would be to conduct plaque assays on viral isolates and measure the concentration of infectious virions. Unfortunately, the DENV-3 isolate utilized does not plaque well. This isolate was selected after pilot experiments (also including DENV-2) showed that DENV-3 had the greater ability of replicating in the presence of *Wolbachia*.

Assuming our theory of defective genome copy enrichment in Aag-2*w*Mel-passaged isolates is correct, there are two possible mechanisms behind the population crashes observed during the serial passaging regime. *Wolbachia* may be creating an intracellular environment that interferes with the accurate and efficient replication of the DENV genome, increasing the ratio of defective to non-defective genomes. *Wolbachia* infection in insect cells is known to increase ROS production (Pan et al. 2012; Wong, Brownlie, and Johnson 2015) that negatively affects RNA virus replication (Choi et al. 2004). Alternatively, *Wolbachia*-induced blocking may simply have caused viral population sizes to fall below a threshold necessary to sustain functional complementation, given an unaltered number of defective genome copies constantly present in cell culture (Juarez-Martinez et al. 2013).

Replicated virus in Aag-2*w*Mel-passaged isolates could be the product of low levels of non-*Wolbachia*-infected cells in the cell lines rather than survivors of selection. While *Wolbachia* prevalence rates appeared high via FISH throughout passaging, we cannot rule out the presence of low numbers of uninfected or very lowly infected cells. Viral variants that are the products of these cells, therefore, would not have had the opportunity to face purifying selection imposed by the blocking phenotype. These variants may also outnumber true products of *w*Mel-infected cells and may explain why the Aag-2*w*Mel-passaged isolates did not exhibit adaptation. Care must, therefore, be taken when investigating variant sequences of these isolates, as the consensus sequence may not reflect the adapted variants. Combined with the inability to analyze sequences of Aag-2*w*Mel-passaged isolates, it is difficult to confirm from our study whether evolution has occurred during the serial passaging regime.

Due to obligate replication in two disparate host systems, the adaptive evolution of arboviruses is thought to be slower relative to other RNA viruses transmitted through a single host species. In particular, beneficial mutations in one host may be antagonistic in the alternate host, incurring a fitness tradeoff (Holmes 2006), in turn increasing the strength of purifying selection. Purifying selection has also been found to act at the intracellular and extracellular levels (Stapleford et al. 2014; Grubaugh et al. 2016; Lequime et al. 2016). The presence of *Wolbachia* adds an additional layer of constraint on top of the strong purifying selective forces shaping DENV evolution, considerably impeding its evolutionary potential. Given that half of our Aag-2*w*Mel-passaged isolates experienced population extinction and the surviving half failed to exhibit significant population expansion or fitness gains, our experiment has demonstrated that *Wolbachia*-induced blocking poses a great challenge for DENV-3 (strain 08/09) adaptation.

Regardless, the sheer scale of the release of *Wolbachia* into global populations of mosquitoes significantly amplifies the opportunity for evolutionary events to occur, over time, that could allow the emergence of viral escape variants. Additionally, viral replication in the human host or in a *Wolbachia*-free mosquito (in a mosquito population where *Wolbachia* infection is not fixed) will release the virus from *Wolbachia*-associated constraints and may allow viral genetic diversity to recover. Further investigations into the sequences of full-length viral variants that successfully replicate and escape from *Wolbachia*-infected cells will give insights into which viral genes can confer adaptation to *Wolbachia*-induced blocking. Advice from our experimental regime indicates that researchers wishing to select for DENV escape variants in the presence of *Wolbachia* should significantly increase the scale of replication on our design, increase the length of serial passaging, and/or utilize cell lines infected with *Wolbachia* strains that exhibit weaker blocking than *w*Mel. Additionally, starting with an ancestral virus isolate of a high titer (in the range of 10^9^ genome copies per µl) will not only mimic field conditions more closely, but also increase the probability of escape variants emerging.

## Data availability

All raw data are available via Figshare: http://doi.org/10.26180/5c2f68d897005. Sequence data have not been made available given poor coverage of the genome.

## Supplementary Material

vez012_Supplementary_Data
