# Supplementary material for: Sustained Wolbachia-mediated blocking of dengue virus isolates following serial passage in Aedes aegypti cell culture
Source: Virus Evol. 2019 Jun 8;5(1):vez012. doi: 10.1093/ve/vez012 (PMC6555872; doi:10.1093/ve/vez012)
Supplement: vez012_Supplementary_Data [file vez012_supplementary_data.zip › Supplementary Figure 1.docx]

**
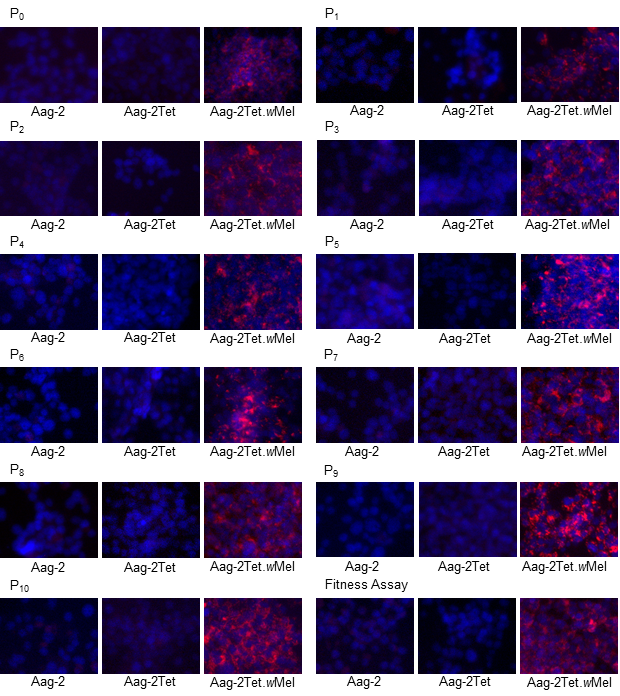
**

**Supplementary Figure 1. FISH images of Aag-2, Aag-2Tet, and Aag-2wMel cells.** *Wolbachia* (stained red) is present in Aag-2*w*Mel cells at every passage, whereas Aag-2 and Aag-2Tet cells are *Wolbachia*-free. Cell nuclei are stained blue.
