# Supplementary material for: Sustained Wolbachia-mediated blocking of dengue virus isolates following serial passage in Aedes aegypti cell culture
Source: Virus Evol. 2019 Jun 8;5(1):vez012. doi: 10.1093/ve/vez012 (PMC6555872; doi:10.1093/ve/vez012)
Supplement: vez012_Supplementary_Data [file vez012_supplementary_data.zip › Supplementary Table 1.docx]

**Supplementary Table 1. Primers and probes used in qRT-PCR assays**

| **Target** | **Primer/probe name** | **Sequence 5’ -> 3’** |
| --- | --- | --- |
| *WD0513* in *w*Mel | TM513_Fwd | CAAATTGCTCTTGTCCTGTGG |
|  | TM513_Rev | GGGTGTTAAGCAGAGTTACGG |
|  | TM513_LTC640N | LTC640N-TGAAATGGAAAAATTGGCGAGGTGTAGG - IAbRQSp |
| *rps17* in *Ae. aegypti* | RPS17_Fwd | TCCGTGGTATCTCCATCAAGCT |
|  | RPS17_Rev | CACTTCCGGCACGTAGTTGTC |
|  | RPS17_FAM | FAM-CAGGAGGAGGAACGTGAGCGCAG - BHQ1 |
| DENV 3’UTR | DENV_Fwd | AAGGACTAGAGGTTAGAGGAGACCC |
|  | DENV_Rev | CGTTCTGTGCCTGGAATGATG |
|  | DENV_FAM | FAM-AACAGCATATTGACGCTGGGAGAGACCAGA-BHQ1 |
